# Five-Year Marginal Bone-Level Changes Around Immediately Loaded or Immediately Restored Implants After Maxillary Sinus Floor Elevation: A Retrospective Cohort Analysis of Three Implant-Site Preparation Techniques

**DOI:** 10.3390/diagnostics16193169

**Published:** 2026-09-29

**Authors:** Cristian Niky Cumpătă, Paolo Di Francesco, Mădălina Anca Moldovan, Denisa Alexandra Al Share, Mihaela Jana Țuculină, Călin Rareș Roman

**Affiliations:** 1 Department of Oral and Maxillofacial Surgery, Faculty of Dental Medicine, Titu Maiorescu University, 031593 Bucharest, Romania; nikycumpata@yahoo.com; 2 Department of Prosthodontics, Faculty of Dental Medicine, Titu Maiorescu University, 031593 Bucharest, Romania; dr.difrancescopaolo@gmail.com; 3 Department of Oral and Maxillofacial Surgery and Implantology, Faculty of Dental Medicine, “Iuliu Hațieganu” University of Medicine and Pharmacy, 400012 Cluj-Napoca, Romania; raresroman25@gmail.com; 4 Dental Clinic, 1 Burebista Blvd., 031106 Bucharest, Romania; 5 Department of Endodontics, Faculty of Dental Medicine, University of Medicine and Pharmacy of Craiova, 200349 Craiova, Romania; mtuculina@yahoo.com

**Keywords:** dental implant, maxillary sinus floor elevation, osteotome, osseodensification, Densah, DASK Simple, marginal bone level, immediate restoration, immediate loading

## Abstract

**Background/Objectives**: Peri-implant marginal bone stability is a relevant indicator of the medium-term outcomes of implants placed simultaneously with maxillary sinus floor elevation. This study aimed to describe peri-implant marginal bone levels after at least 60 months and to assess their association with three implant-site preparation and condensation techniques: osteotomes, Densah/Versah burs, and DASK Simple, used in conjunction with transcrestal or lateral sinus floor elevation. **Methods**: This retrospective cohort study included 248 patients and 528 Dentium SuperLine bone-level implants placed in the posterior maxilla. Immediate restoration without occlusal contacts was used for single-unit or splinted prostheses, whereas immediate loading was applied exclusively to All-on-X rehabilitations. Marginal bone levels were assessed by cone-beam computed tomography (CBCT) at delivery of the definitive restoration, 3–6 months after implant placement, and at the final follow-up, 63–66 months after implant placement. The primary analysis used a linear mixed-effects model to account for dependence among implants within the same patient. **Results**: Six documented implant losses occurred before the final assessment, representing 1.1% of the implants in the selected cohort. Paired radiographic measurements were available for 522 implants. The mean marginal bone level at the final assessment was 0.11 ± 0.18 mm, and the mean change from baseline was 0.03 ± 0.06 mm. Inter-examiner reproducibility was excellent (ICC = 0.994, 95% CI, 0.992–0.996), with a standard error of measurement (SEM) of 0.0085 mm and a 95% minimum detectable change (MDC95) of 0.0235 mm. Repeated intra-examiner measurements showed no numerical differences at the recorded measurement precision. The mean longitudinal change remained substantially below the nominal 0.125-mm CBCT voxel size and should therefore be interpreted cautiously with respect to measurement precision and clinical relevance. After adjustment for the available covariates and clustering of implants within patients, the implant-site preparation and condensation technique was not independently associated with the 5-year marginal bone level. **Conclusions**: No adjusted association was detected between implant-site preparation technique and the 5-year marginal bone level in this retrospective cohort. The very small observed longitudinal changes should be interpreted cautiously in view of measurement uncertainty, potential selection bias, and residual confounding, and the findings should not be interpreted as evidence of equivalence among the three techniques.

## 1. Introduction

Implant-supported rehabilitation of the atrophic posterior maxilla is frequently limited by reduced residual bone height resulting from post-extraction alveolar ridge resorption and maxillary sinus pneumatization [1]. Maxillary sinus floor elevation is used to increase the vertical subantral bone volume and facilitate dental implant placement in the posterior maxilla [1,2]. The procedure involves elevation of the Schneiderian membrane and creation of a space between the sinus floor and the membrane, with or without an augmentation material, depending on the surgical indication and residual bone height [1,2].

Sinus floor elevation can be performed through a lateral or crestal/transcrestal approach [1,3]. The lateral approach provides direct visual access to the lateral sinus wall and is used primarily in cases with substantial vertical deficiency [1]. The transcrestal approach is considered less invasive but does not provide direct visualization of the Schneiderian membrane and depends on local anatomy, residual bone height, and intraoperative control of the Schneiderian membrane [1,3]. Selection of the approach should consider not only residual bone height but also anatomical factors such as maxillary sinus contour, the presence of antral septa, and the condition of the Schneiderian membrane [1].

Systematic reviews evaluating maxillary sinus floor elevation through lateral or transalveolar approaches have reported favorable implant survival outcomes but have highlighted substantial heterogeneity in surgical protocols, augmentation materials, residual bone height, and follow-up duration. Outcomes of the transalveolar approach are influenced by case selection, available bone height, and primary implant stability. Analyses of osteotome-mediated techniques have confirmed predictable outcomes but have not consistently demonstrated the superiority of a particular technical variant or the use of grafting material [4,5,6,7,8,9,10,11,12,13,14].

The osteotome technique is one of the conventional methods of transcrestal maxillary sinus floor elevation [15]. It uses progressive bone condensation and transmission of a controlled axial force to the sinus floor, resulting in indirect elevation of the Schneiderian membrane [15]. The literature indicates that osteotome-mediated sinus floor elevation can yield favorable outcomes, including in selected situations without grafting material; however, the long-term evidence remains dependent on case selection and the surgical protocol used [2].

The need for routine use of an augmentation material in transcrestal sinus floor elevation remains debated. Maintenance of the space beneath the Schneiderian membrane by the implant and stabilization of the blood clot may permit bone formation even without grafting material. Systematic reviews and prospective studies have reported high implant survival rates and intrasinus bone gain for both grafted and graft-free procedures. Nevertheless, these results should be interpreted in relation to residual bone height, implant length, membrane elevation height, and patient-selection criteria [9,10,16,17,18,19,20,21,22].

Osseodensification was introduced as an osteotomy preparation technique based on bone preservation and compaction, in contrast to conventional subtractive drilling [23]. When dedicated burs are used in the prescribed mode, osseodensification densifies the osteotomy walls and can also be applied during indirect transcrestal sinus floor elevation [23,24]. A recent meta-analysis comparing osteotome-mediated transcrestal sinus floor elevation with osseodensification reported similar clinical and radiographic outcomes for bone gain but emphasized that the number of available osseodensification studies remains limited [24]. Another 2025 synthesis concluded that transcrestal osseodensification provides implant survival comparable to that reported for osteotome techniques and lateral-window augmentation in short-term studies, while indicating that randomized trials with longer follow-up are required before the superiority of any technique can be supported [25].

The biological rationale for osseodensification is non-subtractive osteotomy preparation, with compaction of bone particles toward the walls of the implant site. Experimental studies have reported increased peri-implant bone density, primary stability, and bone-to-implant contact in low-density bone [26,27,28]. Clinical application of osseodensification burs for transcrestal sinus floor elevation has produced favorable radiographic and implant outcomes in prospective and randomized studies [29,30,31]. However, most of these studies included relatively small samples and shorter follow-up periods than the present study; therefore, the available evidence does not establish the long-term superiority of osseodensification over the osteotome technique.

Procedure selection should also consider surgical morbidity, the risk of Schneiderian membrane perforation, and available therapeutic alternatives in cases of reduced residual bone height. Transcrestal approaches may reduce surgical invasiveness, but membrane control is indirect and perforations may be difficult to identify intraoperatively [32,33,34,35]. In selected cases, short implants with or without limited transcrestal elevation may represent an alternative to extensive augmentation. Meta-analyses have not identified consistent differences in implant survival between short implants and standard-length implants placed in conjunction with sinus floor elevation; however, treatment selection should be individualized according to local anatomy and prosthetic requirements [36,37,38].

Assessment of implant success should not be limited to implant survival [39]. Criteria reported in the literature include mobility, pain, peri-implant infection, peri-implant radiolucency, soft-tissue status, and marginal bone loss [39]. The peri-implant marginal bone level is important because it may reflect physiological postoperative remodeling but may also serve as a marker of long-term biological or biomechanical risk [39,40]. Therefore, studies evaluating implants placed after augmentation procedures should report not only survival rates but also radiographically measured marginal bone changes [39,40].

The importance of marginal bone loss is also supported by comparative data on implants placed in augmented and pristine bone [40]. A 2025 meta-analysis reported greater mean marginal bone loss at 5 years for implants placed in augmented sites than for implants placed in pristine bone [40]. The same analysis concluded that bone augmentation may be a risk factor for marginal bone loss but is not its sole determinant, highlighting the need to evaluate local and patient-related factors [40]. Consequently, medium- and long-term assessment of bone stability after maxillary sinus floor elevation has direct clinical relevance [40].

Marginal bone loss should be interpreted with reference to both statistical significance and the clinical magnitude of the differences [39,41]. Some studies have proposed early radiographic thresholds, such as marginal bone loss greater than 0.5 mm after loading, as potential indicators of subsequent unfavorable outcomes [41]. However, very small differences, even when statistically significant in large samples, should be interpreted cautiously when the absolute values remain below thresholds considered clinically relevant [39,41].

Technique-specific commercial protocols have also been developed for implant-site preparation during transcrestal sinus floor elevation. The Versah system uses dedicated osseodensification burs within a manufacturer-defined crestal sinus protocol [42], whereas the DASK Simple system incorporates dedicated compaction instrumentation with depth-controlled preparation for sinus-floor management [43].

The unit of analysis is an important methodological consideration in implant research. When the same patient contributes multiple implants, observations cannot be considered fully independent, and the statistical analysis must account for within-patient dependence. In implant survival studies, Chuang et al. showed that including all implants without adjusting for correlations among implants within the same patient may affect estimates of statistical precision and recommended methods that account for clustered observations [44].

In this context, the present study was designed to evaluate peri-implant marginal bone levels 5 years after implants were placed simultaneously with maxillary sinus floor elevation through transcrestal or lateral approaches using three implant-site preparation and condensation techniques: osteotomes, Versah burs, and DASK Simple. Implants were immediately loaded in All-on-X rehabilitations or immediately restored without occlusal contacts in all other situations. The primary objective was to assess the association between the documented implant-site preparation and condensation technique and the 5-year peri-implant marginal bone level within this retrospective cohort. Secondary objectives were to assess changes in marginal bone level from baseline, describe the proportion of implant losses, and determine the distribution of 5-year marginal bone-level values. The statistical dataset comprised 248 patients and 528 implants, and the primary analysis was planned at the implant level using models appropriate for correlated observations.

## 2. Materials and Methods

### 2.1. Study Design and Clinical Setting

A retrospective observational cohort study was conducted based on the clinical and radiographic records of patients who received implant-supported treatment in the posterior maxilla. The study was performed at the Implantology Clinic of the Faculty of Dental Medicine, Titu Maiorescu University, Bucharest, Romania.

Implants were placed between January 2019 and January 2021 simultaneously with maxillary sinus floor elevation through a transcrestal or lateral approach and with implant-site preparation and condensation using one of the three techniques evaluated: osteotomes, Versah burs, or DASK Simple. The implants were immediately loaded or immediately restored after placement, depending on the type of rehabilitation.

All patients were examined, diagnosed, treatment-planned, and surgically treated by the same specialist in oral surgery and implantology.

Patients were reassessed annually. For the present analysis, the CBCT examination performed at delivery of the definitive restoration, 3–6 months after implant placement, was defined as the analytical baseline; the final CBCT examination was obtained at the long-term follow-up, 63–66 months after implant placement.

All baseline marginal bone-level values entered in the analytical dataset were derived from this definitive-restoration CBCT examination; the immediate postoperative CBCT was not used for extraction of the baseline marginal bone-level values included in the statistical analyses.

The database was structured at both the patient and implant levels. The primary analysis was performed at the implant level because baseline marginal bone level, 5-year marginal bone level, membrane elevation height, and implant status were extracted from the clinical charts and operative database individually for each implant. Because the same patient could contribute one or more implants, within-patient dependence was addressed using statistical models for correlated observations.

No a priori sample-size calculation was performed because of the retrospective design. The study size was determined by the number of cases available in the clinical archive that fulfilled the predefined eligibility criteria during the study period.

### 2.2. Eligibility Criteria

The final cohort included 248 adults who underwent placement of one or more implants in the posterior maxilla between January 2019 and January 2021, simultaneously with maxillary sinus floor elevation. Implant sites were prepared using osteotomes, Versah burs, or DASK Simple, and the implants received either immediate loading or immediate provisional restoration.

The clinical indications for maxillary sinus floor elevation and selection between transcrestal and lateral approaches were consistent with established anatomical and surgical considerations reported in the literature [1,3].

The inclusion criteria were as follows: age ≥ 18 years, complete clinical documentation, clear identification of the implant-site preparation and condensation technique, available information on the sinus approach or approaches used at the patient level, and either (i) availability of both the analytical baseline radiographic assessment and the 5-year radiographic assessment for the longitudinal marginal bone-level analysis, or (ii) documented implant loss before completion of the radiographic follow-up for the implant-status analysis.

Cases were excluded when clinical or radiographic documentation was incomplete, neither a 5-year assessment nor documentation of implant loss before that time point was available, information on the sinus approach was insufficient, or the implant-site preparation technique could not be identified. Implants lost before the 5-year radiographic assessment were retained in the implant-status analysis but were not included in the numerical analysis of the final marginal bone level because no valid radiographic value was available at that time point. Implants lost before the analytical baseline examination at definitive restoration were retained only in the implant-status analysis and were not included in the longitudinal marginal bone-level analysis.

### 2.3. Study Groups

Patients were grouped according to the implant-site preparation and condensation technique documented in the operative records:

Group 1 comprised cases in which implant-site preparation and condensation were performed with osteotomes, a conventional bone-condensation approach described for transcrestal sinus floor elevation [15].

Group 2 comprised cases in which implant-site preparation was performed with Densah/Versah burs in osseodensification mode [23,24,25].

Group 3 comprised cases in which implant-site preparation and condensation were performed using the DASK Simple system.

Classification was based on information recorded in the clinical charts and operative database.

The implant-site preparation and condensation technique was the primary exposure variable and was used in both descriptive comparisons and adjusted statistical models.

### 2.4. Recorded Variables

Variables were defined separately at the patient and implant levels.

No standardized questionnaire was administered because the study was based exclusively on retrospective review of existing clinical and operative records.

At the patient level, age, gender, total number of implants, smoking status, presence of chronic periodontitis, and the presence of at least one transcrestal and/or lateral sinus approach were extracted from the available clinical records. Smoking status and chronic periodontitis were recorded according to the information documented in the patients’ clinical charts. The transcrestal and lateral approaches were coded separately as present or absent because the same patient could have implants placed using both approaches. Because these approach variables were available only at the patient level, they could not be reliably assigned to individual implants and were therefore used for descriptive purposes only.

At the implant level, the implant-site preparation and condensation technique, membrane elevation height in millimeters, baseline marginal bone level, long-term marginal bone level, implant status, and immediate prosthetic protocol—immediate restoration without occlusal contacts or immediate functional loading—were recorded.

Residual bone height and width, bone quality, implant-level sinus approach, implant position, individual implant length and diameter in the analytical file, prosthesis type and splinting, and intraoperative complications were not available in a sufficiently complete and standardized implant-level form for multivariable adjustment. All-on-X status was not entered separately because immediate functional loading occurred exclusively in All-on-X rehabilitations and was therefore structurally collinear with the immediate prosthetic protocol.

### 2.5. Radiographic Assessment

Cone-beam computed tomography (CBCT) examinations were obtained at different stages of the clinical treatment and follow-up protocol. The immediate postoperative CBCT examination was clinically indicated for three-dimensional assessment of implant position, the augmented subantral region, and possible procedure-related complications after simultaneous sinus floor elevation and implant placement; however, it was not used as the analytical baseline for marginal bone-level change. For the present analysis, the CBCT examination performed at delivery of the definitive restoration, 3–6 months after implant placement, constituted the analytical baseline. The long-term CBCT examination was obtained at the final follow-up, 63–66 months after implant placement, and constituted the final radiographic assessment and the primary radiographic outcome.

The clinical indication for the long-term CBCT examination was three-dimensional reassessment of the augmented sinus–implant complex, including the augmented subantral region and the spatial relationship between the implant and the maxillary sinus floor, as part of the established radiographic follow-up protocol.

Standardized periapical radiographs are appropriate for routine mesial and distal marginal bone assessment; however, they do not provide the three-dimensional information required to evaluate the augmented maxillary sinus, the subantral region, and the spatial relationship between the implant and the sinus floor. Therefore, the present retrospective analysis used the existing clinically acquired CBCT datasets for marginal bone-level measurements. All values were expressed in millimeters and analyzed individually at the implant level.

Baseline and follow-up CBCT examinations were performed at the same clinic using the same Veraview X800 CBCT system (J. MORITA Corp., Kyoto, Japan). The acquisition protocol used a field of view (FOV) of 80 × 80 mm, a voxel size of 0.125 mm, a tube voltage of 80 kV, a tube current of 6 mA, an exposure time of 9.4 s, and 180° rotation. Patient-specific radiation-dose metrics were not available in the retrospective imaging archive and therefore could not be reported. No additional CBCT examinations were performed specifically for the present retrospective analysis.

A value of 0 mm was recorded when the most coronal radiographically visible bone-to-implant contact coincided with the implant-shoulder reference level, with no measurable apical distance between the two landmarks.

The CBCT datasets were exported in Digital Imaging and Communications in Medicine (DICOM) format and imported into Romexis software, version 6.5.3 (Planmeca Oy, Helsinki, Finland), in which linear measurements were performed. The software-integrated measurement tools used the dimensional information contained in the DICOM volumes. No additional calibration based on the nominal implant length or diameter was applied.

Images were analyzed using multiplanar reconstruction. Before each measurement, the volume was reoriented by aligning the long axis of the implant in the reconstruction planes to obtain comparable sections and standardize the assessment plane between the baseline and final examinations. Measurements were performed on images acquired with the prosthetic abutment in place.

The marginal bone level was determined separately on the mesial and distal surfaces of each implant. For each surface, the linear distance was measured from the implant shoulder to the most coronal radiographically visible bone-to-implant contact. Mesial and distal values were recorded separately, and the implant-level value used in the statistical analyses was calculated as their arithmetic mean [39,40,41].

The change in marginal bone level during follow-up was calculated for each implant as the difference between the value obtained after at least 60 months and the baseline value: Δ marginal bone level = final follow-up marginal bone level − baseline marginal bone level. Positive values indicated apical displacement of the bone-to-implant contact, corresponding to marginal bone loss, whereas negative values indicated an apparent coronal displacement.

Radiographic assessments were performed independently by three clinicians experienced in oral implantology and CBCT interpretation. When discrepancies occurred during the original assessment process, the images were reassessed jointly and the final value was established by consensus. Values were reviewed at the individual implant level. The original radiographic assessments were not performed under a formally documented blinding protocol; therefore, examiner blinding with respect to the study group and assessment time point cannot be confirmed retrospectively.

An additional inter-examiner reproducibility assessment was performed on a stratified random subset of 60 final follow-up CBCT examinations obtained 63–66 months after implant placement, with 20 examinations randomly selected from each implant-site preparation technique. The same examinations were independently assessed by the three clinicians using the marginal bone-level measurement protocol described above. Inter-examiner reliability was evaluated using a two-way random-effects, absolute-agreement, single-measure intraclass correlation coefficient (ICC). Absolute measurement error was quantified using the standard error of measurement (SEM) and the 95% minimum detectable change (MDC95). For the intra-examiner repeatability assessment, each of the three examiners independently repeated the marginal bone-level measurements on the same selected CBCT examinations using the same measurement protocol. The repeat measurements were performed 7–9 days after the initial reproducibility measurements. For each examiner, the repeated measurements were identical to the corresponding initial measurements at the recorded measurement precision, with no numerical differences observed between paired readings.

Exact reconstruction parameters, fixed window-level settings, measurement-display increment, and metal-artifact-reduction settings were not systematically recorded in the retrospective archive and therefore could not be reconstructed reliably.

In this manuscript, values measured at each time point were defined as the marginal bone level, whereas the longitudinal difference represented the change in marginal bone level or marginal bone loss when displacement was apical. The marginal bone level measured at the final follow-up, 63–66 months after implant placement, was the primary radiographic outcome, whereas the change from the definitive-restoration baseline obtained 3–6 months after implant placement was the secondary radiographic outcome [39,40,41].

### 2.6. Retrospective Study Design

The study had a retrospective observational cohort design and was based on clinical and imaging data from the clinic archive. Cases that met all inclusion criteria and none of the prespecified exclusion criteria were included. Cases with incomplete clinical or imaging documentation were excluded before inclusion in the final statistical analysis.

Patients underwent implant placement simultaneously with maxillary sinus floor elevation between January 2019 and January 2021. Immediate loading with functional occlusal contacts was used exclusively in All-on-X rehabilitations. Immediate restoration without occlusal contacts was used for all other restorations. Follow-up included annual assessments and imaging reassessment after at least 60 months.

The final cohort comprised 248 patients and 528 Dentium SuperLine implants (Dentium Co., Ltd., Suwon, Republic of Korea), classified according to implant-site preparation and condensation technique: osteotomes, Densah/Versah burs, or DASK Simple. Each patient was assigned to a single group, and different techniques were not used within the same patient. All techniques were used during the same period.

All procedures were performed by the same specialist in oral surgery and implantology. Technique selection was based on the operator’s clinical judgment, the indications and limitations of each procedure, and the anatomical characteristics of each case. No randomization or prespecified allocation protocol was used for the three groups; therefore, confounding by indication was possible.

Implants lost before reassessment after at least 60 months were excluded from the quantitative analysis of the final marginal bone level because a valid radiographic measurement could no longer be obtained. They were retained in the implant-status analysis and in the calculation of the proportion of lost implants. No formal time-to-event analysis was performed. Therefore, the 1.1% value represents the proportion of documented implant losses within the selected retrospective cohort and should not be interpreted as a 5-year survival rate.

**Figure 1:**
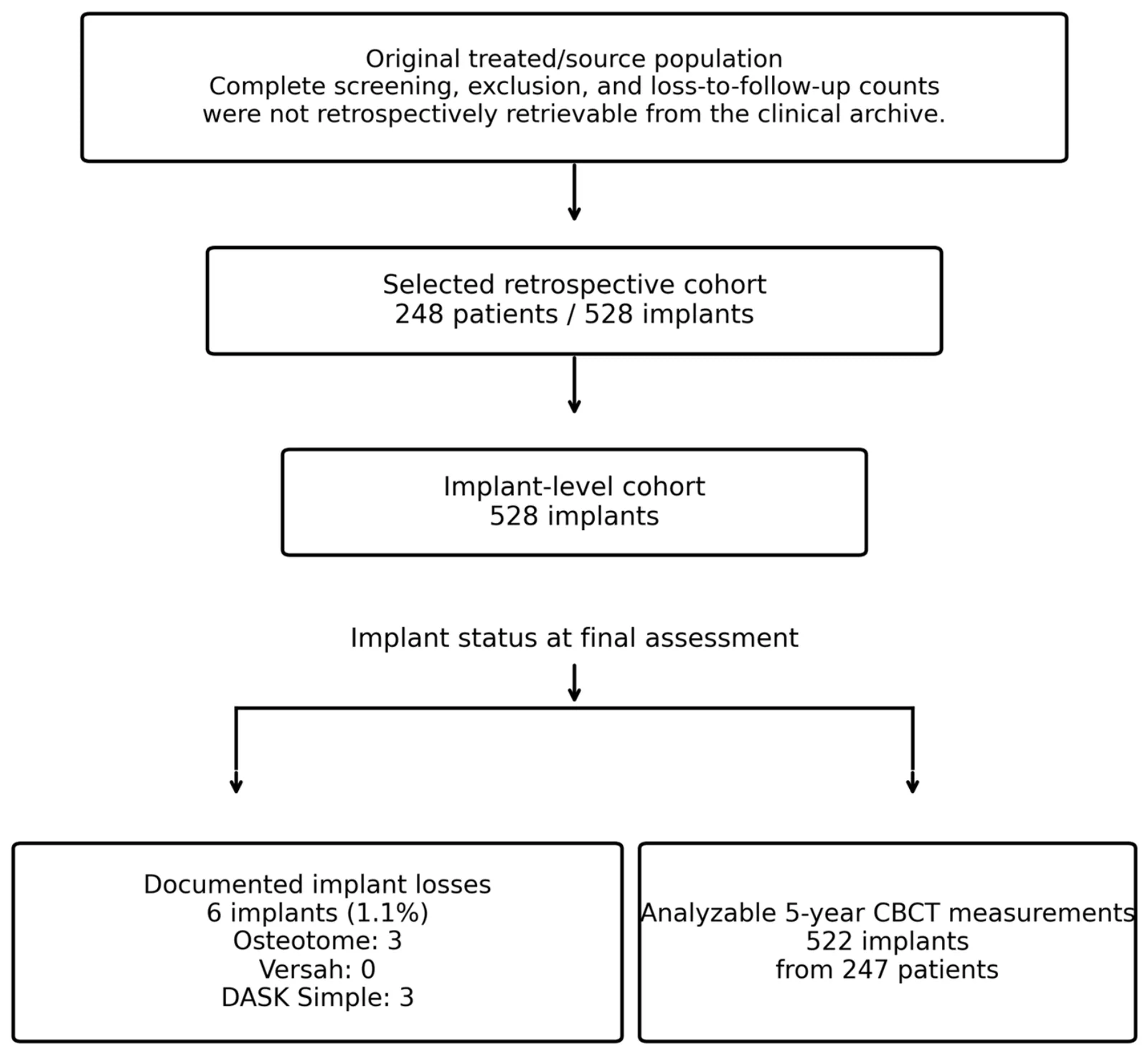
Patient- and implant-level flow of the available retrospective cohort. The complete size of the original treated population and the numbers of exclusions and losses to follow-up could not be retrospectively reconstructed from the clinical archive.

Eligibility criteria were applied uniformly to maintain a consistent cohort definition. However, the total number of patients and implants initially assessed and the distribution of reasons for exclusion could not be retrospectively retrieved. Therefore, the complete source-population flow could not be reconstructed. The available patient- and implant-level flow of the selected retrospective cohort is presented in Figure 1.

### 2.7. Surgical and Prosthetic Protocol

Procedures were planned using preoperative CBCT to assess residual bone height and width, the relationship to the maxillary sinus floor, and the prosthetically guided three-dimensional implant position. All procedures were performed under local anesthesia after elevation of a full-thickness mucoperiosteal flap. The same surgeon applied a consistent operative protocol throughout the study period. Systemic antibiotic prophylaxis was administered preoperatively according to the patient’s allergy status. Amoxicillin was used in patients without penicillin allergy, whereas clindamycin was used as the alternative in patients with a documented penicillin allergy. Immediately before surgery, patients rinsed with 0.2% chlorhexidine gluconate for 60 s.

Selection of the sinus approach took into account a minimum residual bone height of 4 mm, a minimum crestal width of 5 mm, the planned elevation height, and local sinus anatomy [1,3,32,33,34,35]. The transcrestal approach was used when membrane elevation of no more than approximately 3 mm was required. A lateral-window approach was selected when more than approximately 4 mm of elevation was required. These thresholds were clinical selection guides rather than randomized allocation criteria. The same patient could have sites treated through different approaches according to local anatomy.

Assignment to the Osteotome, Versah, or DASK Simple group was determined by the technique used for osteotomy preparation and condensation to achieve the primary stability required for the prosthetic protocol. With the lateral approach, the Schneiderian membrane was elevated through a lateral window and the implant site was prepared using one of the three techniques. With the transcrestal approach, the group-specific technique was used both for osteotomy preparation and for controlled management of the sinus floor.

The implant-site preparation protocol was adapted to the technique used in each group. The implant-site preparation sequence was technique-specific and remained undersized relative to the planned 4.0- or 4.5-mm implant. The purpose was to preserve available bone and obtain primary stability, but the three systems were not treated as mechanically interchangeable: osteotomes compacted bone by serial manual expansion, Densah burs used the proprietary osseodensification protocol, and the DASK Simple compaction drill used its own reverse-rotation mechanism. In all cases, the osteotomy diameter was kept smaller than the nominal diameter of the planned implant to preserve and compact peri-implant bone and achieve primary stability appropriate for immediate restoration or loading.

In the Osteotome group, the rotary osteotomy was initiated and kept undersized using instruments from the Dentium SuperLine Surgical Kit; the final rotary instrument before osteotome use was the 2.6-mm Lindemann First Drill (XLD2635; Dentium Co., Ltd., Suwon, Republic of Korea). Concave osteotomes of progressively increasing diameter were then introduced under controlled axial manual force to enlarge the site and compact trabecular bone laterally and apically. At transcrestal sites, the sinus floor was managed incrementally to limit membrane elevation; at lateral-window sites, membrane elevation was performed under direct visualization before osteotome-assisted site enlargement. The exact osteotome diameter sequence used in individual cases could not be reconstructed retrospectively and was therefore not inferred. The osteotomes allowed controlled enlargement of the site and compaction of trabecular bone toward the osteotomy walls to increase primary stability, consistent with the principles of osteotome-mediated site preparation [15,20,21,22].

In the Versah group, Densah burs were used according to the manufacturer’s crestal sinus protocols and implant-specific drilling chart [42]. With residual bone height ≥ 6 mm, the pilot osteotomy was terminated approximately 1 mm short of the sinus floor; with 4–5 mm of residual height, the manufacturer’s Protocol II avoided a pilot drill. The 2.0-mm Densah bur was advanced in osseodensification mode to the sinus floor, followed by 3.0-mm and, when required, wider burs in counterclockwise rotation at 800–1500 rpm with copious irrigation and a modulating pumping motion. Advancement beyond the sinus floor was performed in 1-mm increments and did not exceed 3 mm. For lateral-window cases, the burs prepared an undersized implant site after direct membrane elevation, without using the crestal apical-advancement step.

Autogenous compaction of bone particles toward the site walls, characteristic of osseodensification, was intended to preserve bone and increase peri-implant bone density [23,26,27,28]. Clinical application of this approach in transcrestal sinus floor elevation has been described previously [29,30,31].

In the DASK Simple group, the initial osteotomy was performed with the 2.2-mm Lindemann Guide Drill (XLD2235) and subsequently with the 3.0-mm Compaction Drill (SRT3035AS), both from Dentium Co., Ltd. (Suwon, Republic of Korea). The compaction drill was used in reverse rotation with a depth stopper selected from the system’s 4–10-mm range; this mechanism compresses bone laterally while guiding autogenous bone chips apically toward the sinus floor [43]. At transcrestal sites, depth-controlled advancement and the autogenous bone-chip buffer were used for limited membrane elevation; at lateral-window sites, the implant bed was prepared after direct membrane elevation. This DASK Simple reverse-compaction protocol is technically distinct from the proprietary Densah osseodensification sequence.

Augmentation material was used with both the transcrestal and lateral approaches. With the transcrestal approach, THE Graft xenograft (Purgo Biologics, Seongnam, Republic of Korea, particle size, 1.0–2.0 mm) was progressively introduced through the osteotomy after membrane elevation, while avoiding excessive compaction. With the lateral approach, the subantral space was augmented using the same xenograft combined with platelet-rich fibrin (PRF). The amount was adapted to the elevation height and anatomy of each site; exact volumes could not be uniformly quantified retrospectively.

Implants were placed simultaneously with sinus floor elevation. Primary stability was assessed using insertion torque and resonance frequency analysis. ISQ was measured with an Osstell device (Osstell AB, Gothenburg, Sweden); the specific device model and SmartPeg type were not systematically recorded. General clinical thresholds for immediate restoration or loading were ≥35 N·cm and ISQ ≥ 60; for single implants, the thresholds were ≥40 N·cm and ISQ ≥ 65. Maximum insertion torque was limited to 60 N·cm. These thresholds were components of the clinical protocol and should not be interpreted as universal loading criteria.

All implants were Dentium SuperLine bone-level implants with an active-thread design, diameters of 4.0 or 4.5 mm, and lengths of 8, 10, or 12 mm. They were placed simultaneously with sinus floor elevation at posterior maxillary premolar or molar sites. Implant position and dimensions were selected prosthetically in relation to available bone, sinus anatomy, and the need for primary stability.

Provisional PMMA restorations were delivered within 24–72 h and were either screw- or cement-retained. Except for splinted All-on-X rehabilitations, provisional restorations were kept out of static and dynamic occlusal contact; All-on-X provisionals received controlled functional occlusion. Definitive restorations were delivered after 3–6 months and consisted of monolithic zirconia restorations or zirconia restorations supported by a titanium framework; they were screw- or cement-retained according to the prosthetic plan.

Small Schneiderian membrane perforations were covered with a collagen membrane [32,33,34,35]. Larger perforations were closed using 6-0 resorbable sutures. Bleeding from the posterior superior alveolar artery was controlled using local hemostatic measures, including infiltration with a local anesthetic solution containing a vasoconstrictor and, when required, direct clamping of the vessel.

Postoperative antibiotic therapy was prescribed according to the same allergy-based protocol. Patients without penicillin allergy received amoxicillin 1000 mg every 12 h for 7 days, whereas patients with a documented penicillin allergy received clindamycin 600 mg every 8 h for 7 days. Ibuprofen 400 mg every 8 h for 3 days was prescribed for postoperative analgesia and inflammation control. Patients rinsed with 0.2% chlorhexidine three times daily for 14 days.

### 2.8. Follow-Up Period

Patients were reassessed annually after implant treatment. The final CBCT examinations were obtained 63–66 months after implant placement, depending on the individual treatment date. The 5-year time point was selected to assess medium-term marginal bone stability and implant retention, consistent with previous long-term evaluations of implants placed in conjunction with maxillary sinus floor elevation [11,12,14,40]. Intermediate annual assessments were part of routine clinical monitoring but were not analyzed as separate statistical time points in the present study.

Among the 522 implants with analyzable final CBCT measurements, the follow-up duration from implant placement to the final CBCT examination was 63.94 ± 1.06 months, with a median of 64 months [63, 65] and a range of 63–66 months. Mean follow-up duration was 63.94 ± 1.10 months in the Osteotome group, 63.89 ± 1.04 months in the Versah group, and 64.02 ± 1.03 months in the DASK Simple group.

### 2.9. Outcome Definitions

The primary outcome was the 5-year peri-implant marginal bone level, expressed in millimeters and analyzed as a continuous variable [39,40].

Secondary outcomes were the change in marginal bone level between baseline and the 5-year assessment, the proportion of implants lost before the final assessment, and the distribution of implants according to the 5-year marginal bone-level value: 0 mm, >0 mm, >0.5 mm, and >1 mm [39,41]. For documented implant losses, the time from implant placement to loss was retrieved from the available records, and the clinical documentation was reviewed for any identifiable cause or procedure-related factor associated with the loss. For descriptive reporting, failures occurring within 6 months after implant placement were classified as early and those occurring thereafter as late.

The change in marginal bone level was calculated as follows: Δ marginal bone level = 5-year marginal bone level − baseline marginal bone level. Positive values indicated marginal bone loss, whereas negative values indicated an apparent coronal displacement of the bone-to-implant contact.

### 2.10. Statistical Analysis

Statistical analyses were performed using IBM SPSS Statistics for Windows, version 32.0 (IBM Corp., Armonk, NY, USA). Quantitative variables were described using the mean, standard deviation, median, interquartile range, and minimum–maximum values, whereas categorical variables were described using absolute and relative frequencies.

For the inter-examiner reproducibility analysis, a two-way random-effects, absolute-agreement, single-measure ICC was calculated and reported with its 95% confidence interval. The 95% confidence interval was estimated by bootstrap resampling of the examinations. Absolute measurement error was expressed as the standard error of measurement (SEM), and the 95% minimum detectable change was calculated as MDC95 = 1.96 × √2 × SEM. Intra-examiner repeatability was assessed separately for each of the three examiners by comparing the repeated measurements with the corresponding initial measurements on the same CBCT examinations. Paired differences were summarized using the mean paired difference and the maximum absolute paired difference.

Baseline characteristics of the three groups were compared using Pearson’s χ^2^ test or exact tests for categorical variables and the Kruskal–Wallis test for quantitative variables with asymmetric distributions. Because these implant-level comparisons did not account for clustering of implants within the same patient, they were considered descriptive or exploratory, and the primary inference was based on models for correlated observations.

The distribution of the 5-year marginal bone level was assessed using tests of normality and examination of skewness and the proportion of zero values. Given the positively skewed distribution and the high frequency of 0-mm values, unadjusted comparisons among techniques were performed primarily using the Kruskal–Wallis test. Post hoc comparisons used the Mann–Whitney U test with Bonferroni adjustment.

Given the asymmetric distribution and the large proportion of zero differences, the Wilcoxon signed-rank test was used for the paired descriptive comparison between baseline and 5-year marginal bone-level values. Because this implant-level nonparametric analysis did not account for clustering of implants within patients, it was interpreted as descriptive. Longitudinal inference was based on the hierarchical mixed-effects model described below. Model assumptions for the primary linear mixed-effects regression were evaluated using standardized residuals. Residual normality was assessed using Q–Q plots and formal normality tests, whereas homoscedasticity was evaluated using residuals-versus-fitted plots, the Breusch–Pagan test based on fitted values, and Levene’s test across quartiles of fitted values. Because the residual diagnostics indicated departures from normality and homoscedasticity, the Gaussian mixed-effects model was retained for estimation of adjusted differences expressed directly in millimeters but was not used as the sole inferential approach. Its findings were therefore evaluated together with cluster-aware sensitivity analyses adapted to the zero-inflated and positively skewed outcome distribution.

The primary adjusted analysis used a linear mixed-effects regression model estimated by maximum likelihood, with the 5-year marginal bone level as the dependent variable, implant-site preparation and condensation technique as the primary predictor, and a patient-specific random intercept to account for clustering of multiple implants within the same patient. The model was adjusted for age, gender, membrane elevation height, baseline marginal bone level, total number of implants per patient, smoking status, presence of chronic periodontitis, and the immediate prosthetic protocol. These covariates were selected on the basis of their clinical relevance and their availability in the retrospective dataset and were entered simultaneously into the model to reduce confounding of the association between implant-site preparation technique and the 5-year marginal bone level. Residual bone height and width, bone quality, implant-level sinus approach, implant position, individual implant length and diameter, prosthesis type and splinting, and intraoperative complications were not available in a sufficiently complete and standardized implant-level form for inclusion in the adjusted model. Because immediate loading was used exclusively in All-on-X rehabilitations and immediate restoration was used for all other rehabilitations, All-on-X status was structurally collinear with the immediate prosthetic protocol and was not entered as a separate covariate. Accordingly, the adjusted model was interpreted as an association model based on the available covariates rather than as a comparative-effectiveness or causal model.

To directly assess changes over time according to technique while accounting for the hierarchical data structure, a longitudinal mixed-effects model was estimated with repeated measurements nested within implants and implants nested within patients. Random intercepts were specified for patient and for implant nested within patient. Assessment time, technique, and the time × technique interaction were included as fixed effects, with adjustment for age, gender, and membrane elevation height. The time × technique interaction terms were used to assess whether longitudinal changes differed among the three implant-site preparation techniques.

Because of the high proportion of 0-mm values, a two-part sensitivity analysis was performed. In the first part, the 5-year marginal bone-level value was modeled as 0 mm versus >0 mm using a logistic generalized estimating equation (GEE) model with a binomial distribution, logit link function, exchangeable working correlation structure, and patient as the clustering variable. The model was adjusted for age, gender, and membrane elevation height. In the second part, the analysis was restricted to implants with positive 5-year marginal bone-level values (>0 mm). The magnitude of the positive marginal bone-level values was modeled using a Gamma GEE model with a log link and an exchangeable working correlation structure, again with patient as the clustering variable. The Gamma model was selected because these conditional values were strictly positive and positively skewed. Exponentiated coefficients from this model were interpreted as ratios of mean positive marginal bone-level values.

As an additional clinically oriented sensitivity analysis, the >0.5-mm threshold was evaluated separately. This threshold was not derived from the reproducibility analysis and was therefore not interpreted as a measurement-error cutoff. A logistic GEE model with patient-level clustering was estimated for a 5-year marginal bone level > 0.5 mm versus ≤0.5 mm. Because only 22 implants exceeded this threshold, this analysis was considered exploratory and was interpreted cautiously in view of the limited number of events and the resulting imprecision of the estimates.

Lost implants were analyzed separately and reported descriptively according to technique, affected patient, timing, early/late classification, and the available clinical documentation regarding possible causes or procedure-related factors. Because only six implant losses occurred and implant-level observations were clustered within patients, no comparative inferential analysis of implant failure was performed.

The robustness of the conclusions was assessed using a patient-level sensitivity analysis. Implant data were aggregated for each patient, and linear regression models were estimated using heteroskedasticity-consistent HC3 standard errors.

The statistical significance threshold was set at *p* < 0.05. *p*-values were interpreted together with confidence intervals, effect sizes, and the clinical magnitude of differences.

### 2.11. Ethical Considerations

The study protocol was approved by the Ethics Committee of Titu Maiorescu University, Bucharest (Decision No. 3 of 20 January 2017), in accordance with the principles of the Declaration of Helsinki. The approval covered the clinical treatment protocol, radiographic monitoring during follow-up, and the use of clinical and radiographic data for research purposes. The approved radiographic follow-up protocol included the postoperative and subsequent follow-up imaging examinations used in the present study, including the long-term radiographic assessment. Although clinical implementation of the approved protocol began later than initially anticipated, implant treatment in the present cohort (January 2019–January 2021) and the subsequent 5-year radiographic follow-up were conducted under the same approved protocol. Written informed consent was obtained from all participants and covered the complete approved treatment and radiographic follow-up protocol, including the clinically indicated CBCT examinations and the research use of de-identified clinical and imaging data. The CBCT examinations analyzed in the present retrospective study were therefore part of the approved clinical protocol and did not represent additional imaging exposure performed specifically for this retrospective analysis.

## 3. Results

### 3.1. General Characteristics of the Study Cohort

The study included 248 patients who received implants between January 2019 and January 2021 and were monitored annually until the 5-year assessment. All implants were Dentium SuperLine bone-level implants. Patients were grouped according to the implant-site preparation and condensation technique used: 96 in the Osteotome group, 81 in the Versah group, and 71 in the DASK Simple group. General characteristics are presented in Table 1.

The distribution of the number of implants per patient was comparable among groups (*p* = 0.759). No significant differences among techniques were observed for the presence of a lateral approach (*p* = 0.411), presence of a transcrestal approach (*p* = 0.625), smoking status (*p* = 0.387), or chronic periodontitis (*p* = 0.702). These data are presented in Table 1.

### 3.2. Implant Characteristics

The implant-level analysis included 528 Dentium SuperLine bone-level implants. Of these, 78 were immediately loaded as part of All-on-X rehabilitations, and 450 were immediately restored without occlusal contacts. According to the implant-site preparation and condensation technique, 204 implants were included in the Osteotome group, 171 in the Versah group, and 153 in the DASK Simple group. Implant characteristics and marginal bone-level values are presented in Table 2.

Six documented implant losses occurred before the final assessment, representing 1.1% of the 528 implants in the selected retrospective cohort. Three losses occurred in the Osteotome group, none in the Versah group, and three in the DASK Simple group. The six losses occurred in six different patients, with no patient experiencing more than one implant loss. The Osteotome-group losses occurred at 2, 3, and 2 months after implant placement, whereas the DASK Simple-group losses occurred at 3, 1, and 2 months, respectively. Thus, all six losses occurred within the first 3 months after implant placement and were classified as early failures; no late failures were recorded. The available clinical documentation did not permit a definitive case-specific etiologic attribution for each implant loss. Moreover, review of the records did not identify a common clinical or procedure-related factor that could be specifically attributed to the implant-site preparation technique. Given the very small number of events, no etiologic or comparative inference regarding implant failure was attempted, and the findings were reported strictly descriptively. Lost implants were excluded from the numerical analysis of the 5-year marginal bone level but were retained in the implant-status analysis.

Numerical marginal bone-level values were available for 522 implants. Membrane elevation height was similar among techniques, with an overall mean of 3.03 ± 1.26 mm and a median of 3.00 mm [2.00, 4.00]. Mean values were 3.03 ± 1.26 mm for the Osteotome group, 3.04 ± 1.32 mm for the Versah group, and 3.02 ± 1.19 mm for the DASK Simple group, with no significant differences among groups (*p* = 0.997).

### 3.3. Baseline and 5-Year Peri-Implant Marginal Bone Levels

All unadjusted implant-level comparisons reported in this subsection were considered descriptive because they did not account for clustering of implants within patients. Baseline marginal bone levels were low throughout the sample. The overall mean was 0.09 ± 0.15 mm, with a median of 0.00 mm [0.00, 0.20]. Values were nearly identical among groups: 0.09 ± 0.15 mm for Osteotome, 0.09 ± 0.16 mm for Versah, and 0.09 ± 0.15 mm for DASK Simple. The difference among techniques was not statistically significant (*p* = 0.978, Table 2).

At the 5-year assessment, marginal bone levels remained low. The overall mean was 0.11 ± 0.18 mm, with a median of 0.00 mm [0.00, 0.22]. Group means were 0.11 ± 0.17 mm for Osteotome, 0.12 ± 0.18 mm for Versah, and 0.12 ± 0.17 mm for DASK Simple. In the descriptive implant-level comparisons, which did not account for clustering within patients, the Kruskal–Wallis test showed no significant differences among the three techniques (*p* = 0.729). The change in marginal bone level between baseline and the 5-year assessment was small. The overall mean change was 0.03 ± 0.06 mm, with a median of 0.00 mm [0.00, 0.00]. Group-specific mean changes were 0.02 ± 0.06 mm for Osteotome, 0.03 ± 0.06 mm for Versah, and 0.03 ± 0.07 mm for DASK Simple. The Kruskal–Wallis test indicated a statistically significant overall difference (*p* = 0.033, Table 2).

Descriptive post hoc implant-level comparisons of the change in marginal bone level between baseline and the 5-year assessment are presented in Table 3. After Bonferroni adjustment, the difference was significant only between the Osteotome and Versah groups (U = 15,264.5, adjusted *p* = 0.025). Differences between Osteotome and DASK Simple (adjusted *p* = 0.395) and between Versah and DASK Simple (adjusted *p* = 1.000) were not statistically significant.

### 3.4. Distribution of the 5-Year Marginal Bone Level

Among the 522 analyzable implants, 339 (64.9%) had a 5-year marginal bone level of 0 mm. A value > 0 mm was recorded for 183 implants (35.1%). A marginal bone level > 0.5 mm was identified in 22 implants (4.2%), and no implant had a value > 1 mm. These data are presented in Table 2.

In descriptive implant-level comparisons, the proportion of implants with a marginal bone level of 0 mm was 67.2% for Osteotome, 63.2% for Versah, and 64.0% for DASK Simple, with no statistically significant differences among techniques (*p* = 0.693). The proportion of implants with a marginal bone level > 0.5 mm was 3.0% for Osteotome, 6.4% for Versah, and 3.3% for DASK Simple, with no statistically significant difference (*p* = 0.210).

### 3.5. Change in Marginal Bone Level Between Baseline and the 5-Year Assessment

The paired descriptive analysis included 522 implants with values available at both time points. The Wilcoxon signed-rank test indicated a statistically significant difference between baseline and 5-year marginal bone levels (W = 21.0, Z ≈ 8.959, *p* < 0.001). Because this implant-level analysis did not account for clustering of implants within patients, it was interpreted descriptively. Among the analyzed implants, 414 showed no numerical change between baseline and the 5-year assessment, 105 showed an increase, and 3 had a slightly lower value at 5 years. The additional inter-examiner reproducibility analysis included 60 final follow-up CBCT examinations independently assessed by the three examiners. Inter-examiner agreement was excellent, with an absolute-agreement, single-measure ICC of 0.994 (95% CI, 0.992–0.996). The SEM was 0.0085 mm, corresponding to an MDC95 of 0.0235 mm. Paired intra-examiner readings were identical at the recorded measurement precision for each of the three examiners. The mean paired difference and maximum absolute paired difference were both 0 mm for each examiner.

Longitudinal inference was based on the hierarchical mixed-effects model with repeated measurements nested within implants and implants nested within patients. In the Osteotome reference group, the adjusted time effect from baseline to 5 years was 0.020 mm (SE = 0.004, 95% CI [0.011, 0.029], *p* < 0.001). The adjusted longitudinal change of 0.020 mm was substantially smaller than the nominal CBCT voxel size of 0.125 mm. The inter-examiner MDC95 of 0.0235 mm was considered a measure of reproducibility and was not used as a threshold for determining the significance or validity of the longitudinal group-level change. Although the reproducibility assessment demonstrated excellent inter-examiner agreement and complete numerical agreement in the repeated intra-examiner measurements, these analyses quantify measurement reproducibility on the same CBCT datasets and do not establish absolute radiographic accuracy or the full uncertainty associated with longitudinal change between two separate CBCT acquisitions. Therefore, this small statistically significant effect should be interpreted cautiously and should not be regarded as evidence of a clinically meaningful change at the individual-implant level.

The time × technique interactions were not statistically significant. Compared with Osteotome, the additional longitudinal change was 0.008 mm for Versah (SE = 0.007, 95% CI [−0.005, 0.021], *p* = 0.213) and 0.009 mm for DASK Simple (SE = 0.007, 95% CI [−0.005, 0.022], *p* = 0.205). Thus, after accounting for repeated measurements within implants and clustering of implants within patients, the adjusted longitudinal analysis did not detect technique-specific differences in marginal bone-level change over time.

### 3.6. Primary Linear Mixed-Effects Regression Model and Residual Diagnostics

The primary linear mixed-effects model included 522 implants from 247 patients. Only patients represented by at least one implant with a valid numerical 5-year marginal bone-level value contributed to this model. The dependent variable was the 5-year peri-implant marginal bone level, patient was included as a random effect, and the Osteotome technique was used as the reference group. Results are presented in Table 4.

After adjustment for the covariates included in the model, the implant-site preparation and condensation technique was not independently associated with the 5-year peri-implant marginal bone level. Compared with the Osteotome technique, the Versah technique had a coefficient of β = 0.010 (SE = 0.007, 95% CI [−0.004, 0.023], *p* = 0.158). For DASK Simple, the coefficient was β = 0.007 (SE = 0.007, 95% CI [−0.007, 0.021], *p* = 0.301). Baseline marginal bone level was significantly associated with the 5-year marginal bone level (β = 1.057, SE = 0.018, 95% CI [1.021, 1.092], *p* < 0.001). The number of implants per patient showed a significant positive association of small magnitude (β = 0.010, SE = 0.003, 95% CI [0.003, 0.016], *p* = 0.003). In particular, immediate loading, compared with immediate restoration, was not independently associated with the 5-year marginal bone level (β = −0.019, SE = 0.011, 95% CI [−0.040, 0.003], *p* = 0.089).

Age, gender, membrane elevation height, smoking, chronic periodontitis, and the immediate prosthetic protocol did not meet the statistical significance threshold in the adjusted model.

**Figure 2:**
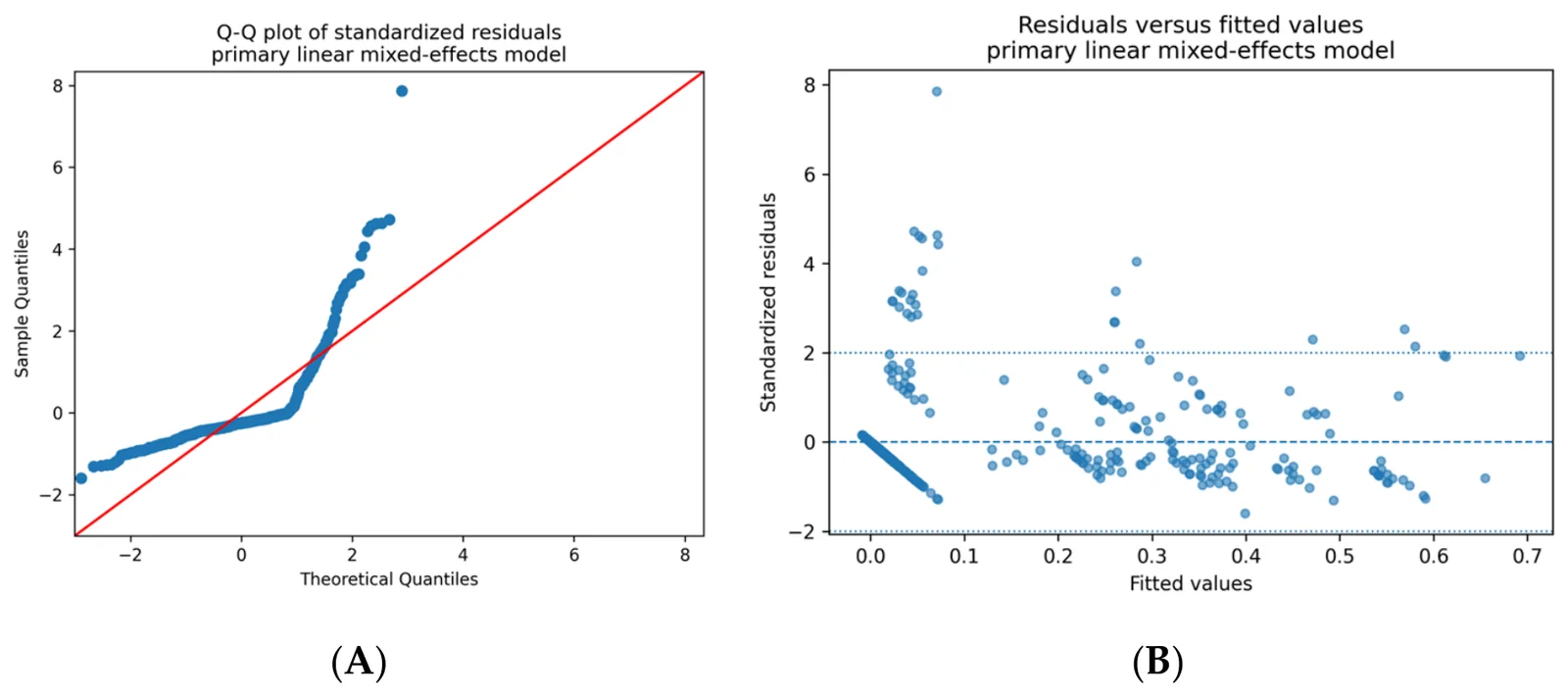
Residual diagnostics for the primary linear mixed-effects model of the 5-year peri-implant marginal bone level. (**A**) Q–Q plot of standardized residuals; the red line represents the theoretical reference line expected under normality. (**B**) Standardized residuals versus fitted values; the dashed horizontal line indicates zero residual, and the dotted horizontal lines indicate standardized residual values of +2 and −2. The Q–Q plot indicates departure from residual normality, whereas the residuals-versus-fitted plot does not show a strong systematic trend.

Residual diagnostics indicated that the assumptions of the Gaussian mixed-effects model were not fully satisfied. Standardized residuals showed a significant departure from normality (Shapiro–Wilk W = 0.662, *p* < 0.001), consistent with the large proportion of 0-mm outcome values (Figure 2A). The residuals-versus-fitted plot did not indicate a strong systematic trend (Figure 2B). The Breusch–Pagan test did not detect global linear heteroscedasticity (LM = 1.032, *p* = 0.310), whereas Levene’s test across quartiles of fitted values indicated differences in residual variance (W = 35.565, *p* < 0.001). Accordingly, the Gaussian mixed-effects model was retained for estimation of adjusted differences directly in millimeters, but its findings were not considered in isolation and were evaluated together with the cluster-aware sensitivity analyses reported below.

### 3.7. Two-Part Sensitivity Analysis of the 5-Year Marginal Bone Level

Because 339 of the 522 analyzable implants (64.9%) had a 5-year marginal bone-level value of exactly 0 mm, a two-part sensitivity analysis was performed. In the first part, the probability of any detectable marginal bone level (>0 mm) was modeled using logistic GEE with patient-level clustering. The unadjusted implant-level comparison of 0 mm versus >0 mm was not statistically significant (χ^2^ = 0.733, *p* = 0.693) and was considered descriptive. The adjusted logistic GEE results for the first part of the analysis are presented in Table 5.

The logistic GEE model, adjusted for age, gender, and membrane elevation height, showed no significant association between technique and the probability of a value > 0 mm at 5 years. Compared with Osteotome, the OR was 1.21 for Versah (95% CI [0.72, 2.02], *p* = 0.478) and 1.10 for DASK Simple (95% CI [0.66, 1.82], *p* = 0.713). Age was not associated with the probability of a marginal bone-level value > 0 mm (OR = 1.00 per additional year, 95% CI [0.98, 1.02], *p* = 0.828). For male versus female gender, the OR was 1.47 (95% CI [0.96, 2.26], *p* = 0.079). Each additional millimeter of membrane elevation was associated with a higher probability of a marginal bone-level value > 0 mm (OR = 1.18, 95% CI [1.02, 1.37], *p* = 0.027).

In the second part of the analysis, the sample was restricted to the 183 implants with positive 5-year marginal bone-level values from 119 patients. Among these implants, the overall mean positive marginal bone level was 0.32 ± 0.14 mm, with a median of 0.31 mm [0.21, 0.41]. Corresponding mean values were 0.33 ± 0.13 mm for Osteotome, 0.32 ± 0.17 mm for Versah, and 0.33 ± 0.12 mm for DASK Simple.

The magnitude of positive marginal bone-level values was modeled using a Gamma GEE model with a log link and patient-level clustering. Implant-site preparation technique was not associated with the magnitude of positive marginal bone-level values. Compared with Osteotome, the estimated mean ratio was 0.98 for Versah (β = −0.022, SE = 0.088, 95% CI [0.82, 1.16], *p* = 0.798) and 1.02 for DASK Simple (β = 0.020, SE = 0.077, 95% CI [0.88, 1.19], *p* = 0.800). Thus, the two-part analysis detected neither an association between technique and the probability of a detectable 5-year marginal bone level nor an association between technique and the magnitude of marginal bone level among implants with positive values.

As an additional clinically oriented sensitivity analysis, the >0.5-mm threshold was evaluated using logistic GEE with patient-level clustering. This threshold was not interpreted as an empirically derived measurement-error cutoff because it was not derived from the reproducibility analysis. Only 22 of the 522 analyzable implants (4.2%) exceeded 0.5 mm. In the adjusted GEE model, technique was not significantly associated with a 5-year marginal bone level > 0.5 mm: the OR was 2.18 for Versah versus Osteotome (95% CI [0.71, 6.73], *p* = 0.175) and 1.21 for DASK Simple versus Osteotome (95% CI [0.30, 4.87], *p* = 0.791). Because of the small number of events and the resulting wide confidence intervals, this threshold analysis was interpreted as exploratory.

### 3.8. Patient-Level Sensitivity Analysis

The patient-level sensitivity analysis was performed by aggregating implant data for each patient. The mean baseline marginal bone level, mean 5-year marginal bone level, maximum 5-year marginal bone level, mean change in marginal bone level, and presence of at least one lost implant were calculated.

In the linear regression model with robust HC3 standard errors for the mean change in marginal bone level, the implant-site preparation and condensation technique was not significantly associated with the mean change in marginal bone level. The coefficient for Versah versus Osteotome was β = 0.006 (95% CI [−0.007, 0.019], *p* = 0.334), and that for DASK Simple versus Osteotome was β = 0.001 (95% CI [−0.012, 0.015], *p* = 0.832).

Results of the patient-level sensitivity analysis are presented in Table 6.

## 4. Discussion

The main findings indicate that peri-implant marginal bone levels remained low at 5 years for all three techniques. The mean value was 0.11 mm, and 64.9% of evaluable implants had a value of 0 mm. Only 4.2% had values > 0.5 mm, and no implant exceeded 1 mm. After adjustment for the baseline value and available covariates, the implant-site preparation and condensation technique was not independently associated with the 5-year marginal bone level.

These findings are consistent with recent syntheses that identified no consistent clinical or radiographic differences between osteotome-mediated transcrestal elevation and osseodensification, although the number of comparative studies and follow-up duration were limited [24,25]. The present study adds a 5-year assessment and includes the DASK Simple technique. However, the observational nature of the cohort precludes the conclusion that the techniques are formally equivalent.

The implant-retention findings are consistent with available long-term data on sinus augmentation procedures. Reviews evaluating follow-up periods of at least 5 years have generally reported high survival rates for both lateral and transalveolar approaches. However, estimates vary according to anatomical characteristics, augmentation material, implant surface, simultaneous or delayed placement, and the definition of implant failure [4,7,9,10,11,12,13,14]. Six documented implant losses occurred in the selected retrospective cohort, corresponding to 1.1% of the 528 included implants. Because the original source cohort and losses to follow-up could not be reconstructed, this proportion should not be interpreted as a formal 5-year survival estimate. However, the six events do not permit a valid comparison of risk among the three techniques.

Marginal bone-level values differed statistically between baseline and the 5-year assessment, but the mean magnitude of the longitudinal change was only approximately 0.03 mm and the median change was 0 mm. The additional reproducibility assessment demonstrated excellent inter-examiner agreement (ICC = 0.994, 95% CI, 0.992–0.996), with an SEM of 0.0085 mm and an MDC95 of 0.0235 mm. Paired intra-examiner readings showed complete numerical agreement at the recorded measurement precision within the assessed sample. This finding does not establish error-free measurement or absolute radiographic accuracy. Moreover, neither the inter- nor intra-examiner assessment fully captures the uncertainty associated with longitudinal differences between two separate CBCT acquisitions. The observed mean change of approximately 0.03 mm also remained substantially smaller than the nominal CBCT voxel size of 0.125 mm. Accordingly, differences on the order of hundredths of a millimeter should continue to be interpreted cautiously with respect to measurement precision and clinical relevance [39,41].

Interpretation of marginal bone changes requires differentiation between physiological remodeling and bone loss associated with peri-implant disease. Current consensus classifications emphasize that peri-implantitis should be identified by integrating clinical signs of inflammation with progressive loss of supporting peri-implant bone [45,46]. Classical and contemporary implant-success criteria include implant retention and the absence of mobility, pain, infection, and radiolucency, as well as marginal bone stability [47,48,49,50]. Epidemiological evidence further supports the need to interpret radiographic bone changes together with standardized clinical assessment when characterizing peri-implant disease [51].

The very low values observed in the present study should also be considered in relation to both measurement precision and the distributional characteristics of the outcome. Implant–abutment connection type, implant geometry, platform position, and the radiographic protocol may influence the apparent marginal bone-level value [49,52,53]. Moreover, approximately two-thirds of the 5-year measurements were exactly 0 mm, and residual diagnostics showed that the assumptions of the Gaussian mixed-effects model were not fully satisfied. The primary model was therefore not interpreted in isolation. In the two-part sensitivity analysis, implant-site preparation technique was associated neither with the probability of a detectable marginal bone-level value (>0 mm) nor with the magnitude of marginal bone level among implants with positive values. Similarly, the hierarchical longitudinal model, which accounted for repeated measurements within implants and implants within patients, did not identify significant time × technique interactions. Therefore, the descriptive unadjusted difference observed between Osteotome and Versah should not be interpreted as evidence of a technique-specific longitudinal effect.

The analysis using the >0.5-mm threshold provided a complementary clinically oriented sensitivity assessment. This threshold was not derived from the reproducibility analysis and should not be regarded as a measurement-error cutoff. Only 22 implants exceeded 0.5 mm, and the cluster-adjusted GEE analysis did not detect a significant association with technique. However, the wide confidence intervals resulting from the small number of events indicate that this finding should be regarded as exploratory rather than confirmatory.

A meta-analysis of studies with long-term follow-up reported greater marginal bone loss in augmented sites than in pristine bone at 5 years [40]. Values in the present cohort were low, but direct comparison with those estimates is not possible because the study did not include a group of implants placed in pristine bone, and definitions of the baseline time point and radiographic protocols may differ among studies.

By the final assessment, six documented implant losses had occurred in six different patients. All occurred within the first 3 months after implant placement and were therefore classified as early failures. Review of the available clinical documentation did not identify a common procedure-related cause attributable to the implant-site preparation technique. However, given the very small number of events, the absence of an apparent technique-related pattern should not be interpreted as evidence of comparable failure risk among techniques. Accordingly, implant losses were evaluated strictly descriptively and no comparative inference was made. The 5-year interval provides a clinically useful window for the cumulative assessment of implant retention and medium-term bone changes.

Smoking and a history of periodontitis are recognized factors that may influence the prognosis of implant treatment and the risk of biological complications [54,55,56,57]. In the present study, chronic periodontitis was not independently associated with the 5-year marginal bone level, and smoking did not meet the statistical significance threshold (*p* = 0.050). Lack of statistical significance should not be interpreted as evidence of no effect, because the retrospective data did not allow quantification of cumulative tobacco exposure, periodontal control, adherence to maintenance therapy, or changes in these factors during follow-up. Their roles in the present cohort should be considered insufficiently clarified rather than refuted.

Baseline marginal bone level showed the strongest association with the 5-year value, as expected in a model adjusted for the starting level. The number of implants per patient showed a positive association of small magnitude. These findings should be interpreted as covariate-adjusted association estimates rather than as a definitive etiological analysis of risk factors.

From a clinical perspective, no independent association was detected between implant-site preparation and condensation technique and the 5-year marginal bone level after adjustment for the available covariates. This result is plausible because outcomes of implants placed in the posterior maxilla are influenced simultaneously by sinus anatomy, residual bone height, primary stability, implant characteristics, the prosthetic protocol, and peri-implant monitoring [8,32,33,45,49]. Therefore, the absence of adjusted differences among techniques does not demonstrate their equivalence in all clinical situations; rather, it indicates that, under the selection and treatment conditions of this cohort, none of the three procedures was associated with a higher 5-year marginal bone level.

The influence of the drilling sequence itself on crestal bone remodeling has also been investigated. Bratu et al. compared a standard five-drill sequence with a shortened three-drill sequence in a paired design involving implants placed in posterior mandibular D2 bone and reported similar crestal bone remodeling at 12 months, while the shortened protocol reduced the time required for implant-site preparation [58]. These findings support the broader biological concept that reducing the number of drills does not necessarily increase early crestal bone remodeling. However, that study involved posterior mandibular D2 bone, conventional implant-site preparation, and a 12-month follow-up, without maxillary sinus augmentation or osseodensification. Therefore, it provides relevant biological context for implant-site preparation but is not directly comparable with the present 5-year cohort of implants placed simultaneously with maxillary sinus floor elevation [58].

Strengths of the study include the relatively large cohort of 528 implants, annual follow-up, the 5-year assessment, assessment of three techniques within the same retrospective cohort, and use of models that account for clustering of implants within patients. The consistency of the findings across the primary linear mixed-effects model, the hierarchical longitudinal model, the two-part cluster-aware analysis, the exploratory > 0.5-mm GEE analysis, and the patient-level sensitivity analysis supports the robustness of the principal conclusion despite the non-Gaussian outcome distribution. Accounting for clustered observations is important in implant studies because ignoring within-patient dependence may lead to inappropriate estimates of precision [44].

In the adjusted model, immediate loading was not independently associated with the 5-year marginal bone level. However, immediate loading was used exclusively in All-on-X rehabilitations; therefore, this result represents adjustment for the prosthetic protocol rather than an independent comparison between immediate loading and immediate restoration.

The study has several limitations. Its retrospective design did not permit complete control of technique selection or confounding by indication. Residual bone height and width, bone quality, implant-level lateral versus transcrestal approach, implant position, individual implant length and diameter, prosthesis type and splinting, and intraoperative complications were not available in a sufficiently complete and standardized implant-level form for inclusion in the adjusted models. Although the immediate prosthetic protocol was included, All-on-X status was not modeled separately because immediate loading was used exclusively in All-on-X rehabilitations. Furthermore, because the sinus approach was available only at the patient level, a reliable implant-level stratified analysis or technique-by-approach interaction analysis could not be performed. Consequently, residual confounding cannot be excluded, and the adjusted findings should be interpreted as descriptive associations within this cohort rather than as estimates of comparative effectiveness.

The small number of lost implants limited analysis of this outcome. The marginal bone-level distribution included numerous 0-mm values, and residual diagnostics indicated departures from normality and evidence of nonconstant residual variance across fitted-value strata in the Gaussian mixed-effects model. Although the principal conclusion was consistent across alternative cluster-aware sensitivity analyses, the distributional limitations and the very small absolute differences warrant cautious interpretation. The additional reproducibility assessment demonstrated excellent inter-examiner agreement, and the repeated intra-examiner measurements showed no numerical differences at the recorded measurement precision. Nevertheless, these assessments were performed on repeated readings of the same CBCT datasets and therefore do not quantify the complete uncertainty associated with longitudinal measurements obtained from separate CBCT acquisitions. Performance of all procedures by the same surgeon reduced interoperator variability but may limit generalizability to other operators and clinical settings.

Exclusion of cases without a final CBCT assessment and without documented implant loss may have introduced selection bias because availability of 5-year imaging may be associated with adherence to follow-up and clinical outcomes.

Within these constraints, the data do not support a clinically relevant difference in 5-year marginal bone level among Osteotome, Versah, and DASK Simple. Technique selection should continue to depend on local anatomy, surgical indications, and operator experience rather than on an assumption of superiority demonstrated by this cohort [1,3].

## 5. Conclusions

In this retrospective cohort, no adjusted association was detected between implant-site preparation and condensation technique and the 5-year peri-implant marginal bone level, and the adjusted longitudinal analysis did not detect technique-specific differences in change over time. The overall longitudinal change was very small and should be interpreted cautiously in relation to measurement precision and clinical relevance. Although inter-examiner agreement was excellent and the repeated intra-examiner measurements showed complete numerical agreement at the recorded measurement precision, the mean longitudinal change of approximately 0.03 mm remained substantially below the nominal CBCT voxel size of 0.125 mm and should therefore be interpreted cautiously. The findings should also be interpreted in the context of potential selection bias and residual confounding inherent to the retrospective design and the incomplete availability of relevant implant-level covariates. Documented implant losses were uncommon in the selected cohort but were too few to support comparative inference. Accordingly, these results describe associations within the selected retrospective cohort and should not be interpreted as evidence of equivalence or comparative effectiveness among the three techniques.

## Figures and Tables

**Table 1:** General characteristics of the patients included in the study.

Variable	Overall Sample (*n* = 248)	Osteotome (*n* = 96)	Versah (*n* = 81)	DASK Simple (*n* = 71)	*p*
Number of patients	248	96	81	71	—
Age, years: mean ± SD, median [min–max]	42.00 ± 10.66, median, 42 [21–67]	41.30 ± 10.25, median, 41 [21–63]	43.68 ± 11.22, median, 46 [21–65]	41.01 ± 10.46, median, 41 [21–67]	0.185
Gender: Female	156 (62.9%)	60 (62.5%)	55 (67.9%)	41 (57.7%)	0.431
Gender: Male	92 (37.1%)	36 (37.5%)	26 (32.1%)	30 (42.3%)	
Implants per patient: 1	80 (32.3%)	35 (36.5%)	25 (30.9%)	20 (28.2%)	0.759
Implants per patient: 2	88 (35.5%)	28 (29.2%)	32 (39.5%)	28 (39.4%)	
Implants per patient: 3	48 (19.4%)	19 (19.8%)	14 (17.3%)	15 (21.1%)	
Implants per patient: 4	32 (12.8%)	14 (14.5%)	10 (12.3%)	8 (11.3%)	
Lateral approach present	98 (39.5%)	33 (34.4%)	34 (42.0%)	31 (43.7%)	0.411
Transcrestal approach present	187 (75.4%)	74 (77.1%)	58 (71.6%)	55 (77.5%)	0.625
Smoking status: Smoker	77 (31.0%)	25 (26.0%)	27 (33.3%)	25 (35.2%)	0.387
Smoking status: Nonsmoker	171 (69.0%)	71 (74.0%)	54 (66.7%)	46 (64.8%)	
Chronic periodontitis: Yes	47 (19.0%)	19 (19.8%)	13 (16.0%)	15 (21.1%)	0.702
Chronic periodontitis: No	201 (81.0%)	77 (80.2%)	68 (84.0%)	56 (78.9%)	

Note. Data are presented as *n* (%) for categorical variables and as mean ± standard deviation and median [minimum–maximum] for age. The Kruskal–Wallis test was used for age, and Pearson’s χ^2^ test was used for categorical variables. The variables “lateral approach present” and “transcrestal approach present” were treated separately as presence/absence variables because the same patient could have implants treated through both approaches.

**Table 2:** Implant characteristics and peri-implant marginal bone-level values.

Variable	Overall Sample	Osteotome	Versah	DASK Simple	*p*
Total number of implants, *n* (%)	528 (100.0%)	204 (38.6%)	171 (32.4%)	153 (29.0%)	–
Lost implants, *n* (%)	6 (1.1%)	3 (1.5%)	0 (0.0%)	3 (2.0%)	–
Implants with analyzable radiographic measurements, *n* (%)	522 (98.9%)	201 (98.5%)	171 (100.0%)	150 (98.0%)	–
Membrane elevation height, mm, mean ± SD	3.03 ± 1.26	3.03 ± 1.26	3.04 ± 1.32	3.02 ± 1.19	0.997
Membrane elevation height, mm, median [Q1, Q3]	3.00 [2.00, 4.00]	3.00 [2.00, 4.00]	3.00 [2.00, 4.00]	3.00 [2.00, 4.00]	
Membrane elevation height, mm, min–max	1.00–6.00	1.00–6.00	1.00–6.00	1.00–6.00	
Baseline marginal bone level, mean ± SD	0.09 ± 0.15	0.09 ± 0.15	0.09 ± 0.16	0.09 ± 0.15	0.978
Baseline marginal bone level, median [Q1, Q3]	0.00 [0.00, 0.20]	0.00 [0.00, 0.20]	0.00 [0.00, 0.14]	0.00 [0.00, 0.20]	
Baseline marginal bone level, min–max	0.00–0.62	0.00–0.53	0.00–0.62	0.00–0.53	
5-year marginal bone level, mean ± SD	0.11 ± 0.18	0.11 ± 0.17	0.12 ± 0.18	0.12 ± 0.17	0.729
5-year marginal bone level, median [Q1, Q3]	0.00 [0.00, 0.22]	0.00 [0.00, 0.21]	0.00 [0.00, 0.22]	0.00 [0.00, 0.26]	
5-year marginal bone level, min–max	0.00–0.80	0.00–0.72	0.00–0.80	0.00–0.72	
Change in marginal bone level, 5 years − baseline, mean ± SD	0.03 ± 0.06	0.02 ± 0.06	0.03 ± 0.06	0.03 ± 0.07	0.033
Change in marginal bone level, 5 years − baseline, median [Q1, Q3]	0.00 [0.00, 0.00]	0.00 [0.00, 0.00]	0.00 [0.00, 0.01]	0.00 [0.00, 0.00]	
Change in marginal bone level, 5 years − baseline, min–max	−0.01–0.51	−0.01–0.51	−0.01–0.31	−0.01–0.33	
5-year marginal bone level = 0 mm, *n* (%)	339 (64.9%)	135 (67.2%)	108 (63.2%)	96 (64.0%)	0.693
5-year marginal bone level > 0 mm, *n* (%)	183 (35.1%)	66 (32.8%)	63 (36.8%)	54 (36.0%)	0.693
5-year marginal bone level > 0.5 mm, *n* (%)	22 (4.2%)	6 (3.0%)	11 (6.4%)	5 (3.3%)	0.210
5-year marginal bone level > 1 mm, *n* (%)	0 (0.0%)	0 (0.0%)	0 (0.0%)	0 (0.0%)	–

Note. For quantitative variables, *p*-values were obtained using the Kruskal–Wallis test. For categorical variables, *p*-values were obtained using Pearson’s χ^2^ test. The marginal bone-level analysis included only implants with available numerical values; lost implants were analyzed separately. IQR, interquartile range, SD, standard deviation.

**Table 3:** Post hoc comparisons of the change in marginal bone level between baseline and the 5-year assessment.

Comparison	Mann–Whitney U	Unadjusted *p*	Bonferroni-Adjusted *p*
Osteotome vs. Versah	15,264.5	0.009	0.025
Osteotome vs. DASK Simple	14,132.5	0.132	0.395
Versah vs. DASK Simple	13,407.0	0.352	1.000

**Table 4:** Linear mixed-effects regression model for the 5-year peri-implant marginal bone level.

Predictor	β Coefficient	SE	95% CI	*p*
Intercept	0.011	0.007	[−0.003, 0.025]	0.133
Versah vs. Osteotome	0.010	0.007	[−0.004, 0.023]	0.158
DASK Simple vs. Osteotome	0.007	0.007	[−0.007, 0.021]	0.301
Age, years	−0.000	0.000	[−0.001, 0.000]	0.425
Male vs. female gender	0.010	0.006	[−0.001, 0.022]	0.086
Membrane elevation height, mm	−0.005	0.004	[−0.013, 0.003]	0.221
Baseline marginal bone level	1.057	0.018	[1.021, 1.092]	<0.001
Number of implants per patient	0.010	0.003	[0.003, 0.016]	0.003
Smoker vs. nonsmoker	−0.012	0.006	[−0.024, 0.000]	0.050
Chronic periodontitis, yes vs. no	0.002	0.009	[−0.016, 0.021]	0.806
Immediate loading vs. immediate restoration	−0.019	0.011	[−0.040, 0.003]	0.089

Note. The dependent variable was the 5-year peri-implant marginal bone level. Patient was included as a random effect, and β coefficients represent the estimated change in the 5-year marginal bone level, expressed in millimeters, associated with the indicated category or with a one-unit increase in the continuous predictor. The Osteotome technique, female gender, nonsmoking status, absence of chronic periodontitis, and immediate restoration were the reference categories. Age, membrane elevation height, and number of implants per patient were centered in the model.

**Table 5:** Logistic GEE model for a 5-year marginal bone-level value > 0 mm.

Predictor	β (Logit Scale)	SE	OR	95% CI	*p*
Versah vs. Osteotome	0.187	0.263	1.21	[0.72, 2.02]	0.478
DASK Simple vs. Osteotome	0.095	0.258	1.10	[0.66, 1.82]	0.713
Age, per year	0.002	0.010	1.00	[0.98, 1.02]	0.828
Male vs. female gender	0.385	0.219	1.47	[0.96, 2.26]	0.079
Membrane elevation height, per mm	0.167	0.075	1.18	[1.02, 1.37]	0.027

Note. OR, odds ratio, CI, confidence interval. The model used a binomial distribution with a logit link and an exchangeable working correlation structure, with patient as the clustering variable. Osteotome and female gender were the reference categories.

**Table 6:** Patient-level sensitivity analysis for the mean change in marginal bone level.

Predictor	β	SE	95% CI	*p*
Intercept	−0.003	0.012	[−0.028, 0.021]	0.789
Versah vs. Osteotome	0.006	0.007	[−0.007, 0.019]	0.334
DASK Simple vs. Osteotome	0.001	0.007	[−0.012, 0.015]	0.832
Age, years	−0.000	0.000	[−0.001, 0.000]	0.384
Male vs. female gender	0.008	0.006	[−0.004, 0.020]	0.180
Mean membrane elevation height per patient	0.002	0.003	[−0.003, 0.007]	0.401
Mean baseline marginal bone level per patient	0.074	0.029	[0.018, 0.131]	0.010
Number of implants per patient	0.008	0.003	[0.002, 0.013]	0.012

Note. Patient-level implant data were aggregated before analysis. Linear regression was estimated using heteroskedasticity-consistent HC3 standard errors. Osteotome was the reference category. β coefficients are expressed in millimeters.

## Data Availability

The original contributions presented in this study are included in the article. Further inquiries can be directed to the corresponding authors.
